# The advantages of metalenses over diffractive lenses

**DOI:** 10.1038/s41467-020-15972-9

**Published:** 2020-04-24

**Authors:** Jacob Engelberg, Uriel Levy

**Affiliations:** Department of Applied Physics, The Faculty of Science, The Center for nanoscience and nanotechnology, The Hebrew University Jerusalem, 91904 Jerusalem, Israel

**Keywords:** Nanophotonics and plasmonics, Applied optics, Metamaterials, Sub-wavelength optics

## Abstract

Optical elements play a crucial role in many modern systems, from cellphones to missiles. The miniaturization trend poses a challenge to optics, since classical lenses and mirrors tend to be bulky. One way of dealing with this challenge is using flat optics. For many years flat optics has been implemented using diffractive optics technology, but in the last two decades a new technology called metasurfaces has emerged. This technology does not replace diffractive optics, but rather expands on it, leveraging the new ability to manufacture subwavelength features on optical substrates. For imaging and focusing applications, diffractive lenses and metalenses are used, as a subset of diffractive optics and metasurfaces, respectively. Recently there has been debate over whether metalenses offer any real advantages over diffractive lenses. In this commentary we will try to gain some insight into this debate and present our opinion on the subject.

## What are metalenses?

The term ‘metamaterial’, referring to subwavelength-level artificially engineered 3D material with desired effective bulk optical parameters, was coined around the year 2000. Metasurfaces and metalenses were invented even before that^1–3^, although these terms, which derive from viewing them as metamaterials with dimensionality reduced to 2D, were introduced more than a decade later^4^. Metalenses have recently gained popularity, with many papers published on this subject in leading journals^5,6^.

**Fig. 1:**
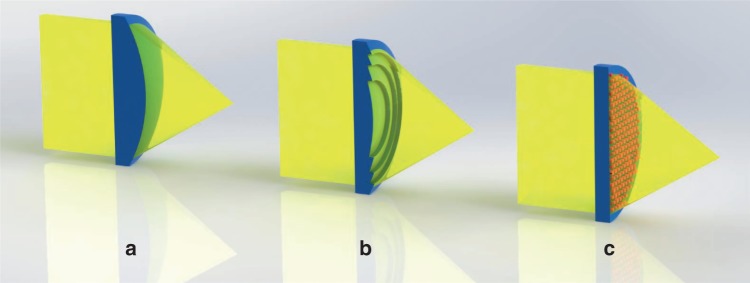
Artistic cross-sectional sketch of lens types. **a** Conventional bulky refractive lens **b** Diffractive lens showing flattening by division into radial zones **c** Metalens showing nanoantennas for phase control.

If we take the example of a positive (focusing) lens, a diffractive lens is an optical element that mimics a plano-convex refractive lens (Fig. 1a), but in which the convex surface is “flattened” by breaking it down into radial zones (Fig. 1b). The penalty is strong chromatic aberration, i.e., at wavelengths other than the design wavelength the focal point shifts linearly with the inverse of the wavelength. From a physical optics point of view one can say that the phase delay in a diffractive lens is introduced modulo 2π (or a multiple thereof). For the purpose of this commentary we define a diffractive lens by this phase wrapping feature, i.e., the phase is limited to a maximal value, thus producing a quasi-periodic structure.

**Fig. 2:**
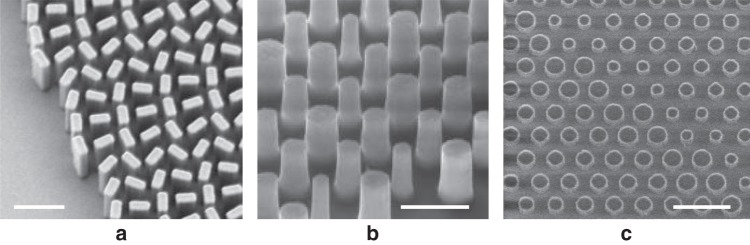
Scanning electron microscope (SEM) images of several metalens antenna forms. **a** Nano-fins used for geometrical phase, reproduced with permission from ref. ^9^, Copyright 2016 AAAS. (**b**) Nano-rods used as truncated waveguides, reproduced with permission from ref. ^7^, Springer Nature and **c** Nano-disks used for Huygens metalens, reproduced with permission from ref. ^12^, De Gruyter. Scale bar is 1 µm.

In a metalens, which is a type of metasurface, the phase is induced via the response of nanostructures (called nanoantennas) built on the surface of the substrate material (Fig. 1c). This contrasts with a conventional diffractive lens (CDL) where the phase inducing mechanism is still like that of a refractive lens—based on the length of the ray path inside the lens material. Three main methods have been used to introduce the phase delay in dielectric metasurfaces: Truncated waveguide^3,7^, geometrical phase^8–10^, and more recently resonant or Huygens^11,12^ nanoantennas (Fig. 2).

Since the phase induced by the nanoantennas is limited in magnitude to about 2π, a metalens of any significant optical power can be considered as a diffractive lens, as it also induces phase modulo 2π. Note that the classical definition for optical power of a lens is the inverse of the focal length, but here we prefer to relate to power as the Fresnel number of the lens, which is the maximum induced phase (of the “unwrapped” wavefront) in units of π. The Fresnel number gives a far more accurate indication of how much “work” the lens is doing in redirecting the flow of light.

We have established that a metalens is a type of diffractive lens, albeit nonconventional in its physics of phase accumulation. However, not every diffractive lens is a metalens. For a lens to be considered a metalens it must have a subwavelength quasi-periodic structure, as opposed to a CDL which is based on a super-wavelength quasi-periodic structure.

Consider the more general case of a metasurface: What can metasurfaces do that conventional diffractive surfaces cannot? As an example, metasurfaces can manipulate the polarization of light, while diffractive surfaces cannot. Metasurfaces can also show resonant behavior, i.e., their properties can be a strong function of wavelength. As such, it is now well established that metasurfaces possess unique properties that cannot be achieved by conventional diffractive surfaces^13^. Yet, going back to the more specific case of a metalens, it is typically only the phase of the light that is manipulated. As this can seemingly be done equally well by a diffractive lens, the advantage of a metalens as compared to a CDL is a subject of debate. Recently, Banerji et al. have taken the position that anything metalenses can do—diffractive lenses can do equally well or better^14^. Metalenses have claimed numerous advantages over CDLs. In the following sections we will look at these individually.

## Reduced thickness

There are questions over whether there is a significant advantage to a ∼100 nm layer (characteristic of a metalens) compared to a ∼1 µm layer (characteristic of a diffractive lens), since they are both constructed on top of a much thicker substrate, typically ∼1 mm thick^14^. This is certainly true from the end-user point of view. From a manufacturing point of view, there is an advantage to thinner structures, but often what determines the manufacturing challenge is the aspect-ratio (height to width) of the structures, which is not necessarily lower in metalenses than in CDLs.

### Binary structure

It is claimed by some in the diffractive community that the manufacture of a multilevel diffractive lens (i.e., a surface profile consisting of several discrete surface heights) is easier than that of a binary (two-level surface height) metalens, as the feature size of the diffractive lens is larger^14^. On the other hand, the metalens community claims that it is simpler to manufacture a binary metalens structure than a multilevel diffractive^15^. It seems to us that “easy” and “hard” to manufacture are subjective terms that depend on the equipment and expertise available to the specific research group or fabrication facility. Clearly both approaches are challenging.

### CMOS compatibility

From a materials point of view, CMOS compatibility is not a distinguishing factor, as there are metalenses that are not CMOS compatible (such as TiO_2_ based), and CDLs that are (such as Si based). Generally speaking, the dimensions of a metalens (small transverse dimensions and depth) are more compatible with a CMOS fabrication line, whereas those of CDLs (larger transverse dimensions and depth) are more compatible with a MEMS fabrication line. Potentially, both can be manufactured in large scales and at low cost. CMOS compatibility is important for any lens that is intended to be incorporated in the manufacturing process of a sensor or other on-chip system that is manufactured using CMOS technology. However, with current advances in wafer stacking technology, the lens wafer can be stacked on the photo sensor wafer at the 12″ wafer scale, so the advantage may not be so crucial.

### High-numerical aperture capability

It has been claimed that high-NA diffractive lenses were demonstrated more than a decade ago, and therefore metalenses offer no advantage in this respect^14^. However, the demonstrated lens^16^ was a binary (two-level) diffractive, and the efficiency of the lens was not reported (the theoretical limit being 40.5%). In addition, it is interesting to note that the fabrication of this lens was performed using a process similar to the process that is used for metalenses (SiN deposition, e-beam write and lift-off), so this example does not support the argument that CDLs can give the same performance as metalenses, with a simpler manufacturing approach. Higher efficiencies can be obtained by using a multilevel diffractive element, with a blaze optimized using the rigorous diffraction grating theory^17,18^. However, this raises the following question: Assuming a moderately high NA, say of 0.5, the smallest period of the diffractive lens turns out to be 2*λ* (*λ* being the central working wavelength of the lens). With 4 phase levels, providing efficiency of ∼70%^13^, the minimum feature size is 2*λ*/4 = *λ*/2 –i.e., it is subwavelength. So, is this a CDL or a metalens? Even if one still defines this lens as a CDL (as the subwavelength features are not quasi-periodic), the advantage of easy fabrication as a result of large super-wavelength features cannot be claimed.

As of today, there are demonstrations of metalenses with significant optical power and NA > 0.9^7,9,11^ with good efficiency, whereas equivalent CDLs have not been demonstrated experimentally.

### Chromatic correction

Chromatic correction of diffractive lenses was demonstrated long ago using multi-order diffractive optics^19^. This was achieved by a diffractive lens operating around a high diffraction order *m* (∼10) instead of the usual first order, which allows several wavelengths to be focused at the same plane. The price paid is the zone depth, which is *m* times larger than that of a standard first-order diffractive. This method has been extended, using numerical optimization methods, to obtain maximum performance at minimum increase in etch depth^14^.

In metalenses, there have been many recent demonstrations of chromatic correction. The most common method used is dispersion engineered nanoantennas^20^. The advantage of the method is that it provides color correction over a continuous wavelength range. However, the method is limited to small optical powers and/or wavelength ranges^20^. Other methods of chromatic correction demonstrated in metalenses are cascading^21^ and transverse multiplexing^22^. These methods can provide correction at several discrete wavelengths, typically at the expense of efficiency.

As chromatic correction can be achieved using diffractive optics, one may argue there is no point in exploring it in metalenses. However, the mechanisms for providing the chromatic correction are physically different. In addition, the number of degrees of freedoms available in a metalens design to tackle chromatic aberrations is much higher than in CDLs. Therefore, we think it is too early to say that metalenses cannot provide better polychromatic performance than what has been achieved with CDLs.

Regarding chromatic correction, we would like to point out that the methods used in both CDLs and metalenses are not scalable, i.e., the fact that good correction was achieved with a small metalens (e.g., 10–100 µm aperture/focal length), does not mean that this concept can be applied to the same metalens when scaled up (to 1–10 mm aperture/focal length). All the achromatic metalenses demonstrated to date are small, thus there is still room for innovation in this field.

### Tunability

There have recently been many demonstrations of tunable metasurfaces and metalenses. In the case of metalenses this is important mostly for focus adjustment, but perhaps also relevant for creating a zoom effect or correcting aberrations. Some of the tuning methods, such as mechanical stretching^23^, may also be applicable to diffractive lenses. However, some are applicable only to metalenses^24^. It seems that metalenses have an advantage in this respect.

### Polarization selectivity

Polarization sensitivity is manifested mostly in geometrical phase based metalenses (where the induced phase is defined by the orientation of otherwise identical nanostructures) and is generally looked upon as a drawback, as it reduces efficiency for unpolarized light. However, in some cases it can be exploited to focus right- and left-circularly polarized light at different lateral^6^ or longitudinal^8^ positions. In principle, it is possible to design various polarization-sensitive functionalities with metalenses. This degree of freedom is not found in CDLs.

### Outlook

In summary, it is true that many metalens applications that have been demonstrated could be achieved equally well with CDLs. Nevertheless, metalenses have already outperformed CDLs in some respects (for example, high-efficiency high-NA applications^7,9^). Metalenses have more degrees of freedom than CDLs, as their nanostructures come in unlimited forms. There is still room for innovation in CDLs, and metalens research should not overshadow this. However, is seems inevitable that the more mature field of diffractive optics, which has already been successfully transferred to industry, will take a backseat in academic research with respect to the newer field of metasurfaces and metalenses, which has yet to prove itself in the field.

Many applications have been suggested for metalenses, in the fields of imaging, spectroscopy, color/polarization routing, tunable focusing, and augmented reality. There may be still unexplored advantages in terms of applications for metalenses. We believe that eventually metalenses will make it into industry, when the appropriate application meets commercial manufacturing capability. However, we do not think they will replace diffractive lenses, since for many applications CDLs are sufficient and more cost effective. As of today, one of the stages metalenses must traverse on the path to commercialization is adoption of good engineering practice, for example the use of relevant performance figures of merit that will allow comparison of different types of lenses. We conclude that the importance of the research does not depend on what buzzword is used, but on its contribution to scientific understanding and potential industrial applications.
